# Parenting and depressive symptoms among adolescents in four Caribbean societies

**DOI:** 10.1186/1753-2000-6-31

**Published:** 2012-09-21

**Authors:** Garth Lipps, Gillian A Lowe, Roger C Gibson, Sharon Halliday, Amrie Morris, Nelson Clarke, Rosemarie N Wilson

**Affiliations:** 1 Department of Sociology, Psychology and Social Work, The University of the West Indies, Mona, Jamaica; 2 Department of Community Health and Psychiatry, The University of the West Indies, Mona, Jamaica; 3 Ministry of Health and the Environment, Government of St. Kitts and Nevis, Basseterre, UK; 4 Ministry of Health, Government of St. Vincent and the Grenadines, Kingstown, UK; 5 School of Clinical Medicine and Research, The University of the West Indies - Bahamas, Nassau, Bahamas

**Keywords:** Parenting, Caribbean nations, Depressive symptoms, Adolescents

## Abstract

**Background:**

The strategies that parents use to guide and discipline their children may influence their emotional health. Relatively little research has been conducted examining the association of parenting practices to depressive symptoms among Caribbean adolescents. This project examines the association of parenting styles to levels of depressive symptoms among adolescents in Jamaica, the Bahamas, St. Kitts and Nevis, and St. Vincent.

**Methods:**

Adolescents attending grade ten of academic year 2006/2007 in Jamaica, the Bahamas, St. Vincent, and St. Kitts and Nevis were administered the Parenting Practices Scale along with the BDI-II. Authoritative, Authoritarian, Permissive and Neglectful parenting styles were created using a median split procedure of the monitoring and nurturance subscales of the Parenting Practices Scale. Multiple regression analyses were used to examine the relationships of parenting styles to depressive symptoms.

**Results:**

A wide cross-section of tenth grade students in each nation was sampled (n = 1955; 278 from Jamaica, 217 from the Bahamas, 737 St. Kitts and Nevis, 716 from St. Vincent; 52.1% females, 45.6% males and 2.3% no gender reported; age 12 to 19 years, mean = 15.3 yrs, sd = .95 yrs). Nearly half (52.1%) of all adolescents reported mild to severe symptoms of depression with 29.1% reporting moderate to severe symptoms of depression. In general, authoritative and permissive parenting styles were both associated with lower levels of depressive symptoms in adolescents. However, the relationship of parenting styles to depression scores was not consistent across countries (p < .05). In contrast to previous research on Caribbean parenting, caregivers in this study used a mixture of different parenting styles with the two most popular styles being authoritative and neglectful parenting.

**Conclusions:**

There appears to be an association between parenting styles and depressive symptoms that is differentially manifested across the islands of Jamaica, the Bahamas, St. Kitts and Nevis and St. Vincent.

## Background

Extensive research has suggested that the strategies which parents use to guide and discipline their children affect their academic, social and emotional development [1-6]. In particular, past research has suggested that parenting practices may influence adolescents’ mental health [2,3,5-7]. Despite this extensive body of research, the majority of studies examine parenting practices and mental health from the perspective of parents with a minority of studies doing so from the perspective of adolescents [8-13]. The current project examines the association between adolescents’ perceptions of parenting and their self-reported symptoms of depression.

### The influence of parenting

Parents are perhaps the most influential persons that one will ever encounter in life. Their impact is both profound and enduring. In fact, parenting styles are largely influenced by parents’ experiences, both positive and negative, with their own parents as they themselves grew up [14]. Other factors such as prevailing cultural norms and expectations [1], religious beliefs [1], and characteristics of the children in the parent–child relationship [15] also shape the dynamic and complex phenomenon of parenting.

In order to better understand parenting and its influence on the lives of children, some efforts have been made to deconstruct the inherent complexities and to categorize parenting styles into discrete entities which may be predictive of certain outcomes. Baumrind [1] was a pioneer in this area and first described three common prototypes of adult control of children: Permissive, authoritarian and authoritative. Over the years this has evolved into four categories of parenting styles – permissive, authoritative, authoritarian, and neglectful - each with its own implications for psychological and behavioural outcomes in children and adolescents.

The four parenting styles are determined by parents’ position along two spectra: low vs. high warmth and low vs. high control [15]. Authoritative parents are generally warm with a high level of positive/ assertive control; authoritarian ones have low warmth and high levels of punitive control; permissive parents are warm but with little attempt at control; and neglectful parents have low warmth and exert low control. Most descriptions of parenting in the Caribbean identify authoritarian parenting as the dominant style [4,16,17]. These reports highlight the common practices of physical punishment and public humiliation as preferred methods of discipline in Caribbean settings [14,16]. It has been hypothesized that these potentially harmful practices have fostered behavioural problems in children and according to Smith and Mosby [14], create a vicious cycle of violence and the perpetuation of punitive, authoritarian parenting.

Apart from implications for conduct problems, authoritarian parenting has also been linked to depression, anxiety, cognitive problems and substance abuse [5,7]. In contrast, adolescents who have experienced authoritative parenting appear to have lower levels of depressive symptoms, as well as higher levels of achievement and competence, when compared with other adolescents and these phenomena are sustained over time [6,18]. Although more so for somatic than for psychological symptoms of depression in adolescents, Steinberg et al. [18] described associations between depression and neglectful and permissive parenting practices. Apart from low levels of adjustment, adolescents from neglectful homes also had relatively high levels of somatic complaints. In contrast, adolescents from permissive homes demonstrated a tendency towards a fair level of adjustment on psychosocial, cognitive and behavioral parameters [18] as well as relatively low levels of somatic or psychological symptoms of depression.

In addition to the apparently central role of level of adjustment in the associations between neglectful and permissive parenting and high and low levels of depression respectively, a number of other mechanisms may be operating in the relationships between parenting style and depression. For example, Smith et al. [7] argue that punitive parenting may cause affected youth to internalize their reactions and that this becomes manifested as negative psychological outcomes. They also theorize that a perception of parental rejection may mediate negative psychological outcomes in this context. In contrast, authoritative parenting is believed to foster a heightened sense of self efficacy and self worth which may be an important mediating factor of the association between this parenting style and low levels of depression. It should be noted that research studying the longitudinal impact of parenting style on adolescents is suggestive of parenting style preceding, as opposed to accompanying or following, adolescent adjustment issues [18].

Any linkages between parenting style and adolescent depression must also be considered in the context of normal adolescent development as well as prevailing cultural norms. In adolescence, the cognitive development that occurs allows young people to shift roles and to take more responsibility for managing their relationships, including those with their parents. Having now to deal with complex emotional issues may be overwhelming and may predispose adolescents to depression, particularly if the emotional issues are related significantly to negative experiences [19]. To a large extent, the outcome of this emotional metamorphosis depends on the prevailing cultural expectations and the extent to which the adolescent’s reality matches these norms. For example, Steinberg et al. [18] found that the negative consequences of authoritarian parenting were less intense among minority groups in North America when compared with persons of European-American descent. They suggest that this may be because adolescents’ interpretations of parental behavior depend on their cultural heritage and suggest as an example that what is interpreted as parental intrusiveness in one cultural context may be interpreted as parental concern in another.

### The epidemiology of depressive symptoms among Caribbean adolescents and its impact

Depression is a leading cause of morbidity worldwide [20] and its presence may significantly erode the productivity of a society [21]. By and large, English-speaking Caribbean countries are of middle income status [22] with optimal social development yet to be achieved. Such development depends on the drive and vitality of current and future generations and becomes threatened when depression is present to a significant degree in young people. Bringing depression under control therefore has implications for both the health of the individual as well as for the well-being of the wider society. Comprehensive strategies to deal with depression will depend on the targeting of crucial causal factors.

Previous research in the Caribbean has indicated that the prevalence of depression in adolescents is high (24.5% to 40.6% prevalence of moderate to severe depressive symptoms) with gender, demographic, socioeconomic and academic factors all being implicated [23-26]. However, there may be other equally important, but yet unexplored, factors associated with depression in this population. Given the reportedly high prevalence of both adolescent depression and authoritarian parenting in the Caribbean as well as the fact that the two phenomena have shown significant association with each other in previous research, the authors hypothesized that in a sample of Caribbean adolescents, depression and authoritarian parenting would be found to be highly prevalent and that they would show a significant association with each other. If this is found to be true, the implication would be that strategies to discourage authoritarian parenting (and to encourage the more adaptive authoritative style) could prove useful in significantly reducing the burden of youth depression in the Caribbean.

The specific objective of the study was to determine the distribution of parenting styles and the extent to which parenting styles are correlated with depressive symptoms. Therefore we hypothesize that:

1. Adolescents in this study will report relatively high levels of depressive symptoms, with females reporting greater symptoms than males.

2. Authoritative parenting styles will be associated with lower levels of depressive symptoms in adolescents.

3. Caribbean parents will tend to use authoritarian parenting practices.

## Methods

### Sample

Grade ten students from four Caribbean islands - Jamaica, the Bahamas, St. Kitts and Nevis and St. Vincent - were chosen to take part in the research project. All four countries included in this study are English speaking independent nations which are part of the British Commonwealth. They are all constitutional monarchies with democratically elected legislatures that govern each country. Jamaica and the Bahamas gained their independence in the early 1960’s while St. Vincent and St. Kitts and Nevis gained their independence in the late 1970’s and early 1980’s respectively. In terms of size and demographic features, Jamaica is 10,991 square kilometres in size, with a population of approximately 2,700,000 people. The majority of the population are of African descent (91%), while 6% are of mixed race and the remainder are of unknown or other ethnicities [27]. The Bahamas is 13,880 square kilometres in size. It has a population of approximately 347,000 people, with 85% of the population of African descent, 12% European and 3% Asian and Hispanic descent [28]. St. Kitts and Nevis is 261 square kilometres in size with a population of approximately 50,000 people who are predominately of African descent with a small number of the population who are European (British, Portuguese, and Lebanese descent) [29]. St. Vincent and the Grenadines is 389 square kilometres in size and has a population of approximately 103,000 people. The majority of the population are of African (66%) or mixed descent (19%), with the remainder either East Asian (6%), European (4%), Carib-Amerindian (2%) or some other ethnicity (3%) [29].

Students attending the tenth grade were chosen as past research has demonstrated that students in the lower grades were more likely to experience emotional problems [30]. Participants expressed strong interest in the study and were highly motivated to take part in the research. As such, no participants refused to take part in the study. However, the study was school-based and youth who were not present on the day of data collection were not included in the sample of each country. Table 1 displays the demographic features of students by gender, maternal education, and age for each country. 

**Table 1:** Demographic features of sampled adolescents

	**Jamaica**		**Bahamas**		**St Kitts**		**St Vincent**	
	**N**	**%**	**N**	**%**	**N**	**%**	**N**	**%**
Gender								
Male	115	41.4	90	41.5	371	50.3	384	53.6
Female	145	52.5	114	52.5	352	47.8	382	46.4
Missing	18	6.5	13	6.0	14	1.9	0	0.0
Maternal Education								
Secondary or less	130	46.8	79	36.4	437	59.9	487	68.0
Post-Secondary	148	53.2	138	63.6	204	27.7	177	24.7
Missing	0	0.0	0	0.0	96	13.0	52	7.3
Age								
13	0	0.0	44	20.3	0	0.0	0	0.0
14	40	14.4	89	41.0	72	9.8	66	9.2
15	179	64.4	64	29.5	309	41.9	342	47.8
16	59	21.2	20	9.2	279	37.9	191	26.7
17	0	0.0	0	0.0	77	10.4	88	12.3
18	0	0.0	0	0.0	0	0.0	29	4.1

#### Jamaica

A cross-section of grade ten students from traditional and non-traditional high schools in urban and rural Jamaica were sampled (n = 278 students; 41% males, 52% females and 7% not stated; age 14 to 16 years, mean = 15.0 yrs ± 0.6 yrs: Table 1). The schools sampled included a traditional urban high school, a non-traditional urban high school, a rural traditional high school and a non-traditional rural high school. This sampling of schools reflects the range and typical diversity of schools found in Jamaica.

Jamaican high schools are stratified into traditional and non-traditional schools. This categorization parallels Jamaica’s socio-economic stratification [31]. Students are assigned to either a traditional or non-traditional high school based on their exit examination performance at the end elementary school. Students who have high scores on that exit examination are selected for attendance in traditional high schools [32]. Attending a traditional high school increases students’ chances of obtaining high grades (e.g. A grades) on the critical competency examinations given at the end of grade eleven. High grades in these exams are needed to move on to university and technical colleges which are the pathways to higher paying and more prestigious occupations [33]. Consequently, students who are placed in non-traditional high schools may be at higher risk of developing depressive symptoms.

#### The Bahamas

Two hundred seventeen adolescents attending the tenth grade were sampled from four high schools on New Providence Island (42% males, 52% females and 6% not stated; age 13 to 16 years, mean = 14.3 yrs ± 0.9 yrs: Table 1). Schools were selected to take part in the project so as to obtain a diverse sampling of schools in various areas of New Providence Island. The school system in the Bahamas is derived from the British colonial system, however it has been influenced to some degree by the North American educational system such that their schools have been organized into school districts which govern each school’s operation [28], with some of the schools being affiliated with the New England Association of Schools and Colleges [34]. In total, there are 252 schools of which 157 are funded by the government with the rest being privately run [35]. Students attending the government funded schools do not pay tuition while those attending the private schools must do so [36].

#### St. Kitts and Nevis

The researchers contacted officials within the Ministry of Education regarding the selection of schools. Because of the high interest in the findings of the study, the Ministry of Education requested that all schools in St. Kitts and Nevis be sampled. As such, a near census of grade ten students attending all high schools in Saint Christopher (St. Kitts) and Nevis were surveyed (n = 744; Table 1). A list of all schools providing secondary education in St. Kitts and Nevis was obtained from the Ministry of Education. Schools were visited and all grade ten students in attendance on that day were surveyed. The sample consisted of nearly equal percentages of female (50.4%) and male (47.6%) students with 2% the sample not reporting their gender. Students in our sample ranged from 13 to 19 years of age (mean = 15.5 yrs ± 0.8 yrs: Table 1). Secondary education is free and compulsory up to grade 11 [37]. As in many Caribbean nations, secondary school is divided into a compulsory 4 year segment, followed by an optional 2 year segment for those students who wish to pursue post-secondary education [37].

#### St. Vincent

Data were collected from 716 grade ten students attending eight secondary schools across the island of St. Vincent (Table 1). A local researcher (APM) selected the schools based on discussions with officials of the Ministry of Education of St. Vincent. Secondary education in St. Vincent is grouped into two forms of schools - traditional and non-traditional high schools with attendance in the traditional high schools being the most sought after [38]. Students are assigned into either a traditional high school or a non-traditional high school based on their performance on a placement examination sat in the last year of primary school [38]. The top 500 students on the exit examination are allowed to choose which traditional high school they attend, while the remainder of students are placed in the non-traditional high school which is closest to where they live [38]. The Ministry of Education suggested non-traditional and traditional high schools to be sampled. Schools were selected such that three traditional and five non-traditional schools were sampled. Of the eight schools sampled, two were boys’ schools, two were girls’ schools and the remaining four were co-educational schools. Students within each school were randomly sampled to take part in the study. Of the 716 participants sampled, 384 (53.6%) were female and 332 (46.4%) were male (Table 1). Students ranged in age between 12 and 19 years (mean = 15.5 yrs ± 1.0 yrs).

### Measures

#### Beck Depression Inventory - II (BDI-II)

The Beck Depression Inventory (BDI-II) [39] is a 21 item questionnaire which examines the cognitive, behavioural, affective and somatic symptoms of depression. Each item of the BDI-II is comprised of a series of rank ordered statements. Each statement is assigned a score from 0 to 3 reflecting the severity of the symptom. Students were asked to circle the number associated with the statement that most accurately describes their feelings during the past two weeks. Previous research suggests that the BDI-II is reliable in North American samples of adults [39]. Studies using both non-clinical [39,40] and clinical samples of adolescents [41] have reported acceptable internal consistency reliabilities, with coefficient alphas ranging from 0.87 to 0.94. Within a sample of Jamaican adolescents the BDI-II had a high internal consistency reliability (*α*= 0.87; [42,43]). In the current sample the BDI-II was found to have a high internal consistency reliability (*α*= 0.88). Past research suggests that the BDI-II is valid across different cultures [39,44], even in cultures that place a high stigma on psychological problems [45]. Based on their BDI-II scores, adolescents in this study were divided into four levels of depressive symptoms - minimal (13 or less), mild (14 to 19), moderate (20 to 28) or severe (29 or higher) symptoms of depression. While depression is a clinical diagnosis requiring professional assessment of adolescents by a clinical psychologist or psychiatrist, the BDI-II simply provides information on levels of depressive symptoms. Some youth, but not all, who have high BDI-II scores may be judged by clinicians to have major depressive disorder.

#### Parenting Practices Scale (PPS)

Parenting practices were measured using Lempers, Clark-Lempers, and Simons’ [5] Parenting Practices Scale. This measure consisted of 29 items whereby adolescents described the strategies their parents used to interact and care for them during the last six months. The PPS was developed using items from Schaefer’s [46] Child’s Report of Parental Behaviour Inventory, items from Roberts, Block and Block’s [47] Child Rearing Practices Report, as well as items Lempers, et al. developed specifically for the measure. The measure was organized into subscales of parental nurturance, parental monitoring and consistency of parental discipline. The measure has an acceptable degree of internal consistency reliability [5] for the measure as a whole (α = 0.80) and for the various subscales (α = 0.76 to 0.81). It has been shown to have some degree of construct validity in that a factor analysis of the items yielded three factors corresponding to the three PPS subscales. The PPS has been used by several researchers to assess parenting [2,48,49].

Four parenting styles were created by using a median split on the parental nurturance and parental monitoring subscales. Students who scored above the median on both parental nurturance and parental monitoring were grouped into an Authoritative parenting style, those who scored above the median on parental monitoring but below the median on parental nurturance were grouped into an Authoritarian style. Those who scored above the median on parental nurturance but below the median on parental monitoring were grouped into a Permissive style while those who scored below the median on both parental nurturance and parental monitoring were grouped into a Neglectful parenting style.

#### Demographic data

A variety of information on students’ demographic features, including their age and gender, was collected using a series of brief questions. Students were asked to report their exact age in years on their last birthday and their gender. Students were also asked to report on their mothers’ highest level of education using the categories of no schooling, kindergarten, primary, secondary/high school, trade/vocational, associate degree, bachelor’s degree and graduate degree. For the analyses these categories were collapsed into secondary education or below or post-secondary education. Maternal education was used as an indicator of social class in some of our analyses^a^.

### Procedure

Prior to the start of the project, a research assistant liaised with the Ministry of Education in each country and the schools selected to participate in the project in order to describe the study’s aims and obtain consent for conducting the project. Students in the classrooms selected for this project were given an informed consent form for their parents to complete. Data for the project were collected from students during one of their regularly scheduled classes. All students in a classroom whose parents provided their informed consent for their adolescent to take part in the project were given a package of instruments to complete. This package consisted of an informed consent form for the adolescents, the BDI-II, the Parenting Practices Scale and a series of questions regarding their demographic features.

### Statistical analyses

Several statistical analyses were conducted. Preliminary analyses were conducted to check and correct data capture problems using simple frequency and cross-tabulations. A check was made on the extent of missing values for each question on the questionnaire. No question was found to be missing more than 10% of responses. On this basis the mean score was substituted for missing values on each question. Basic descriptive statistics were then run to check that the data met the statistical assumptions required by multiple regression analysis. These checks found that the data met the assumptions needed to perform multiple regression. As such, no transformations were made to the data. Multiple regression analyses were conducted to examine the simple associations of gender, maternal education, parenting practices and country with BDI-II depression scores, as well as the interactive association of country with parenting practices. In this regression analysis, BDI-II depression scores served as the dependent variable while gender, parenting practices, country and the interactions of country with parenting practices served as the independent variables. To control in the analyses for the influence of social class, maternal education, was entered into the regression analyses at the first stage of analysis. Dummy coded predictors were used to represent gender (0 = Female, 1 = Male), maternal education (0 = Secondary education or less, 1 = Post-Secondary education), Authoritarian parenting (1 = Authoritarian, 0 = All other types of parenting), Authoritative parenting (1 = Authoritative, 0 = All other types of parenting), Permissive parenting (1 = Permissive, 0 = All other types of parenting), and Neglectful parenting (1 = Neglectful, 0 = All other types of parenting) in the regression analyses. Country of residence was also represented using a series of dummy coded variables with separate predictors created for each country. To aid in the interpretation of interaction effects, statistically significant interactions of country by parenting style were linearlized and a one-way ANOVA, with post-hoc tests was conducted.

## Results

### Prevalence of depression

Simple frequency analyses were conducted to examine the extent of depressive symptoms across the four countries. Nearly half (52.1%) of all adolescents reported mild to severe symptoms of depression with 29.1% reporting moderate to severe symptoms of depression. This prevalence of depression scores however, was not uniform across countries (Table 2). Nearly three-quarters (71.9%) of all adolescents in Jamaica reported mild to severe symptoms of depression with 40.7% reporting moderate to severe symptoms of depression. The prevalence of depressive symptoms among students in St. Vincent was somewhat different from that found in Jamaica because while nearly three-quarters (72.6%) of all adolescents reported mild to severe symptoms of depression only 31.0% of students reported moderate to severe symptoms of depression. Adolescents from St. Kitts and Nevis had the lowest levels of depressive symptoms with only 62.3% of students reporting mild to severe symptoms of depression with 24.7% of students reporting moderate to severe symptoms of depression. The prevalence of depressive symptoms for students from the Bahamas was similar to their peers from St. Kitts and Nevis with 63.3% reporting mild to severe symptoms of depression and 22.6% reporting moderate to severe symptoms.

**Table 2:** Depressive symptoms by country and parenting style

	**Minimal**	**Mild**	**Moderate**	**Severe**
Country				
Jamaica	36.0%	23.4%	26.3%	14.4%
Bahamas	54.4%	23.0%	15.7%	6.9%
St. Kitts and Nevis	53.7%	21.6%	14.0%	10.7%
St. Vincent	44.6%	24.4%	21.8%	9.2%
Parenting style				
Authoritative	60.5%	20.2%	14.2%	5.2%
Authoritarian	34.8%	22.5%	26.3%	16.4%
Permissive	59.3%	22.5%	12.6%	5.5%
Neglectful	35.3%	27.1%	22.8%	14.8%

### Prevalence of parenting styles

Many researchers [3,50] have suggested that Caribbean parents predominately utilize authoritarian parenting. The current study, however, found that adolescents reported their parents used a mixture of different parenting styles. Indeed, the two most popular parenting styles experienced by adolescents were authoritative parenting (32.6%) and neglectful parenting (28.4%). Only 20.3% of adolescents’ parents utilized authoritarian parenting while 18.7% had parents who utilized permissive parenting.

According to adolescents’ reports, parenting styles significantly differed across the four countries (χ^2^(9) = 21.04, p < .01; Table 3). The predominant parenting style in the Bahamas (38.2%), Jamaica (38.1%) and St. Kitts and Nevis (32.7%) was authoritative. In contrast, the predominate parenting style in St. Vincent was neglectful (29.7% of adolescents’ parents).

**Table 3:** Parenting styles by country, gender and maternal education

	**Authoritative**	**Authoritarian**	**Permissive**	**Neglectful**
Country^1^				
Jamaica	38.1%	17.6%	13.3%	30.9%
Bahamas	38.2%	20.7%	16.6%	24.4%
St. Kitts and Nevis	32.7%	18.5%	21.6%	27.3%
St. Vincent	28.6%	23.2%	18.4%	29.7%
Gender^2^				
Females	36.2%	25.8%	13.6%	24.4%
Males	29.2%	14.7%	23.5%	32.5%
Maternal Education^3^				
Secondary or Less	31.7%	20.4%	18.0%	29.9%
Post-Secondary	35.7%	21.6%	18.4%	24.3%

^1^ χ^2^(9) = 21.04, p < .01.

^2^ χ^2^ (3) = 71.84, p < .01.

^3^ χ^2^ (3) = 7.14, p > .05.

Parenting styles also significantly differed by gender (χ^2^ (3) = 71.84, p < .01; Table 3). Female participants reported the highest percentage of parents who utilized authoritative parenting (36.2%) while male participants mainly reported neglectful parenting (32.5%).

Maternal education was not significantly related to parenting styles (χ^2^ (3) = 7.14, p > .05; Table 3). Regardless of their mothers’ level of education, students reported mainly authoritative parenting styles (Table 3).

Authoritarian and neglectful parenting styles were most highly associated with levels of depressive symptoms in all four countries (Table 4). Jamaican students reported the highest levels of depressive symptoms for both authoritarian (mean = 21.18, sd ± 10.58) and neglectful parenting (mean = 21.41, sd ± 9.39) followed by St. Kitts and Nevis students for authoritarian (mean = 19.74, sd ± 11.73) and St. Vincent students for neglectful parenting (mean 18.36 sd ± 10.29). Students from the Bahamas on average reported lower levels of depressive symptoms for authoritarian (mean = 17.00, sd ± 9.86) and neglectful parenting (mean = 17.09, sd ± 9.38).

**Table 4:** BDI-II depression scores by country and parenting styles

	**Parenting Style**				
**Country**	**Authoritative**		**Authoritarian**	**Permissive**	**Neglectful**
Jamaica	Mean	13.77^a^	21.18^abc^	13.00^bc^	21.41^acd^
	sd	10.10	10.58	9.98	9.39
Bahamas	Mean	10.99^bde^	17.00	12.44^bdf^	17.09^eg^
	sd	8.93	9.86	7.19	9.38
St. Kitts	Mean	11.87^bdgh^	19.74^acefhi^	11.40^bdij^	16.32^dehj^
	sd	8.74	11.73	9.18	10.09
St Vincent	Mean	13.36^bdik^	17.53^ehjkl^	13.03^bdilm^	18.36^aehjkm^
	sd	7.95	9.15	9.01	10.29

^a^ Jamaica Authoritative versus others (p < .05).

^b^ Jamaica Authoritarian versus others (p < .05).

^c^ Jamaica Permissive versus others (p < .05).

^d^ Jamaica Neglectful versus others (p < .05).

^e^ The Bahamas Authoritative versus others (p < .05).

^f^ The Bahamas Permissive versus others (p < .05).

^g^ The Bahamas Neglectful versus others (p < .05).

^h^ St. Kitts Authoritative versus others (p < .05).

^i^ St. Kitts Authoritarian versus others (p < .05).

^j^ St. Kitts Permissive versus others (p < .05).

^k^ St. Vincent Authoritative versus others (p < .05).

^l^ St. Vincent Authoritarian versus others (p < .05).

^m^ St. Vincent Permissive versus others (p < .05).

### Regression analyses

Past research has demonstrated that both gender and social class were associated with depression scores [5,24,51-60]. As such, both gender and social class, as measured by maternal education, were used as control variables in the regression analyses. To explore the relationship of country and parenting styles, a three stage hierarchical multiple regression analysis was conducted (Table 4). The regression analysis used parenting styles and country as independent variables and BDI-II scores as the dependent variable. In the first stage of the regression analysis mothers’ highest level of education and students’ gender were entered into the regression equation as control variables. In the second stage of the analysis, country and parenting styles were entered simultaneously into the regression equation. The third stage of analysis entered the interactive effects of country by parenting style. Results of this analysis indicated that there were significant differences in BDI-II depression scores by country, parenting style and the interactions of country by parenting style.

Controlling for social class and gender differences, and using St. Vincent as the comparison group^b^, Jamaican students reported significantly higher overall depressive symptoms (t_(1757)_ = 3.10, p < .01; Table 4) than students in St. Vincent. On average students in Jamaica reported BDI-II scores that were 2.2 points higher than students in St. Vincent. In contrast, controlling for social class and gender, students from the Bahamas (t_(1757)_ = −1.36, p > .05; Table 4) and St. Kitts and Nevis (t_(1757)_ = −1.32, p > .05; Table 4) did not report significantly higher symptoms of depression than students in St. Vincent.

The country by parenting style interactions were statistically significant (R^2^ chg = .01, F(9, 1748) = 2.19, p < .01). To better understand the interactive findings, the country by parenting style interactions were linearized so that post-hoc tests via a one-way ANOVA could be conducted (Table 5). Results of this analysis indicated that several of the interactions of country by parenting style for depressive symptoms were statistically significant (F(15, 1932) = 14.00, p < .01). Jamaican parents who used an authoritative parenting style had adolescents who reported significantly lower BDI-II scores than their peers whose parents used a neglectful parenting style (7.6 points lower), as well as their counterparts whose parents used a authoritarian parenting style (7.4 points lower). Additionally, students of Jamaican parents who used an authoritative parenting style reported significantly lower BDI-II scores (5.8 points lower) than their peers from St. Kitts and Nevis whose parents used an authoritarian parenting style, and students from St. Vincent whose parents used a neglectful parenting style (4.6 points lower).

**Table 5:** Results of regression analyses of country and parenting practices predicting BDI-II depression scores, controlling for gender and maternal education

**Predictor**	**B**	**Beta**	**t**	**Δ R**^**2**^
Stage One – Control Variable				0.06*
Mother’s Education	−1.80	-0.09	−3.70*	
Gender	−4.26	-0.21	−9.08*	
Stage Two – Main Effects				0.09*
Jamaica	2.15	0.08	3.10*	
Bahamas	−1.05	-0.03	−1.32	
St. Kitts	-0.69	-0.03	1.31	
Authoritative Parenting	−5.94	-0.28	−9.59*	
Permissive Parenting	−5.35	-0.20	−7.39*	
Neglectful Parenting	-0.04	-0.00	-0.06	
Stage Three – Interaction Effects				0.01*
Jamaica by Authoritative Parenting	−3.22	-0.08	−1.69	
Jamaica by Permissive Parenting	−4.05	-0.06	−1.74	
Jamaica by Neglectful Parenting	0.90	0.02	0.45	
Bahamas by Authoritative Parenting	−2.07	-0.04	−1.03	
Bahamas by Permissive Parenting	−2.39	-0.03	-0.95	
Bahamas by Neglectful Parenting	-0.07	-0.00	-0.03	
St. Kitts by Authoritative Parenting	−3.88	-0.13	−2.67*	
St. Kitts by Permissive Parenting	−4.68	-0.12	−2.89*	
St. Kitts by Neglectful Parenting	−3.96	-0.12	−2.66*	

* p < .05.

Total variance = .155, Unique variance = .078, Shared variance = .077.

## Discussion

The relationship of parenting styles to depression scores was not consistent across countries. Parenting styles had a stronger relationship to depressive symptoms in Jamaica and St. Kitts than in the other two nations. Authoritative parenting style in St. Kitts was associated with the lowest levels of depressive symptoms. In Jamaica authoritarian and neglectful parenting styles were associated with the highest symptoms of depression. Contrary to previous research [3,50,61] this study found that Caribbean parents did not predominately utilize an authoritarian parenting style. Instead, it appears that they use a mixture of different parenting styles with the two most popular styles being authoritative and neglectful parenting. Further, it appears that the distribution of parenting styles was not consistent across nations.

### Possible explanations for national differences in levels of depressive symptoms

Jamaican adolescents reported the highest levels of depressive symptoms with a large proportion reporting symptoms in the moderate to severe range of depression. This prevelance of depressive symptoms was substantially higher than that found in samples of students in the US (41% :[62], 15%; [63]), in Bosnia & Herzegovina (32%; [64]) or in Italy (18%; [65]), Social and economic inequalities, limited and inadequate infrastructural resources and high rates of violence in Jamaica [66] may explain the possible reasons for such high rates of depressive symptoms in Jamaica. Additionally, the structure and functioning of the educational system may contribute to elevated levels of depressive symptoms. Jamaican adolescents are highly streamed into academic tracks. Past research has found that academic tracking is associated with high levels of depressive symptoms [23].

It is also possible that these findings simply reflect the fact that the largest proportion of adolescents 15 years of age were from Jamaica while the sample from the Bahamas included a larger proportion of adolescents 13 to 14 years of age. It should be noted that the peak prevalence of adolescent depression occurs around 15 years of age [67]. Further, authoritarian parenting may be more detrimental to older adolescents, thus possibly explaining the differences in levels of depressive symptoms by parenting style by country for Jamaica versus the Bahamas.

Students from St. Vincent also reported high levels of depressive symptomatology, although not as high as Jamaican students. The social and economic situation in St. Vincent may contribute to depressive symptoms. Data from CARICOM suggests that there are limited resources and opportunities for citizens of St. Vincent, as well as a high level of unemployment [68]. As such, students from St. Vincent are at increased risk for depression like their peers in Jamaica. Similarly, students in St. Vincent are also academically streamed, thereby possibly contributing to depressive symptoms.

### Prevalence of parenting styles

Literature has often suggested that Caribbean parents use authoritarian parenting styles coupled with harsh disciplinary practices [3,4,7,14,17,69,70]. The current study suggested that parents in the Caribbean may use a variety of parenting styles. For example, in St. Vincent parents tend to use predominately a neglectful parenting style. Practically, this finding suggests that the students often felt that they did not receive much parental monitoring or nurturance. Previous research has suggested that neglectful parenting styles may place adolescents at increased risk for depression [71]. According to the population and housing census 2001 of St. Vincent and the Grenadines [72], many children whose parents had migrated, were parented by extended family members such as grandparents, aunts, uncles and cousins. The report further suggested that the quality of such relationships may have lacked nurturance and some aspects of parental monitoring as well.

Contrary to previous research it appears that adolescents in the Bahamas, Jamaica and St. Kitts and Nevis report that their parents may prefer to utilize an authoritative parenting style. Adolescents who experience this style of parenting reported that they felt that they had received high levels of both parental monitoring and nurturance. It is possible that with the implementation of parenting programs such as the Roving Caregiver Programme in Jamaica, St. Vincent, Dominica and St. Lucia [73] the parenting style may have metamorphosised. The Roving Caregivers Programme provides trained early childhood educators who visit parents and children in their households to educate them in appropriate parenting skills as well as encouraging intellectual development through interactions with both caregivers and children.

Alternatively, research on parenting in the Caribbean has focused on parents’ disciplinary practices, characterizing parents who report they use harsh discipline as having an authoritarian parenting style (e.g. [14]). The current project examines the full range of parenting practices, both positive and negative, allowing for other styles of parenting to emerge. Our approach to assessing parenting styles considers two independent dimensions (nurturance and monitoring) along which childrearing practices may be conceptualized. Under this approach, it is possible for caregivers to utilize a combination of monitoring practices along with differing levels of nurturance. Previous research has focused on parenting styles from the perspective of the caregivers. This project examines parenting practices from the perspective of adolescents. This may help to explain the apparent discrepancy with previous research.

### Parenting and depressive symptoms

While the predominant parenting style was authoritative in this Caribbean study, the authors found that this parenting style, which involves higher levels of warmth as well as monitoring, is associated with adolescents reporting lower levels of depressive symptoms. This is consistent with international literature [6,71]. Further, the permissive parenting style was also associated with lower depression scores. Past research suggests [71] that the high levels of warmth and low levels of monitoring characteristic of permissive parenting leads to less conflict and lower levels of internalizing problems, such as depression and anxiety.

Almost two-thirds of adolescents who experienced authoritative or permissive parenting reported minimal symptoms of depression, while only 5% of these adolescents reported moderate to severe symptoms of depression. This is in sharp contrast to those adolescents who experienced authoritarian or neglectful parenting, both of which involve low levels of warmth and nurturance. In the latter groups, nearly half of all adolescents reported moderate to severe levels of depressive symptoms, further underscoring the importance of parental nurturance as a protective factor against the development of depressive symptoms [71].

The association of parenting styles to depressive symptoms was not consistent among as well as within the Caribbean nations examined. Adolescents who reported that their parents used a neglectful parenting style had higher levels of depressive symptoms generally, with Jamaican adolescents who perceived neglectful parenting reporting the highest differences in depressive symptoms when compared to all other participants. Further, the difference in BDI-II scores for adolescents who reported their parents used either a neglectful or authoritarian parenting style versus an authoritative parenting style was significantly greater in Jamaica than the other three Caribbean nations. These differences in levels of depressive symptoms by parenting styles in Jamaica may be magnified by the socio-economic conditions (e.g.; academic tracking and violence) that exist in Jamaica and that do not exist to the same extent in the other nations.

Adolescents who reported that their parents used an authoritative parenting style did not have similar BDI-II scores across the islands. Teenagers from the Bahamas reported lower BDI-II scores than those from the other three islands. This finding may reflect a interactive relationship between authoritative parenting styles and better socio-economic conditions such as school resources, family income levels, and greater prevalence of two parent families [74].

### Gender and parenting styles

Across the Caribbean, this study found that parenting style was unevenly distributed with respect to gender of the child, such that girls were more likely to receive authoritative parenting styles than their male counterparts. This is consistent with previous research [16,69] which reports that Caribbean parents tend to give more freedom, less parental monitoring and less emotional nurturance to boys [16,69,75]. Previous research has also reported that this pattern of parenting did not hold true for middle class parents who were stated to show more parental monitoring and nurturance to their children regardless of gender.

### Limitations of the research

This project had several key limitations. Data on parenting and depressive symptoms were collected at only one point in time. As such, the cross-sectional design of the study does not allow a causal association of parenting to depressive symptoms to be established. Adolescents reported on the parenting behaviours of their caregivers. Consequently, their reports of parenting may not actually reflect the parenting practices of their caregivers. Similarly, as students completed a self-report measure of depressive symptoms it was not possible to determine if students were clinically depressed. Students were not sampled from all islands comprising the Bahamas or St. Vincent and the Grenadines. Only students in New Providence Island in the Bahamas and St. Vincent were included in this research project. As such, the findings reported here may not be representative of what may be found in the other islands of the Bahamas or the Grenadines. The data for this project were collected from students on only one school day. As such, children who wished to avoid participation in the research may not have attended schools on the day of data collection. However, many of the students expressed strong interest in the research project. Consequently, many of the students were keen to take part in the project.

## Conclusions

The association of parenting styles to depressive symptoms was not consistent across the countries. The unique social conditions present in Jamaica appear to amplify the association of parenting styles to depressive symptoms. The present study found that contrary to previous research, Caribbean parents used a mixture of parenting styles with the predominant method being authoritative. Parenting styles were not homogenous across the Caribbean nations examined. Further, gender appeared to be related to parenting methods. While the authors of this research project tried to offer suggestions for the discrepancies from past research, future research needs to be conducted to more fully describe reasons for these differences. Further, given the high levels of depressive symptoms found in this study, Caribbean parents should be sensitised to symptoms of depression in adolescents and the courses of action which should be followed for further evaluation and assistance of their children. Additionally, parent training in the Caribbean should emphasize the importance of developing and maintaining warm, nurturing relationships with their children as it serves as a protective factor against the development of depression.

## Endnotes

^a^Mothers’ level of education was used as the indicator of social class as most households in the Caribbean are female headed households thereby providing the most complete source of information [45,76].

^b^We chose to utilize St. Vincent as the comparison group because this country had the highest levels of authoritarian and neglectful parenting. From literature [5,7,18] these two parenting strategies have been most strongly associated with depressive symptomatology.

## Competing interests

The authors declare that they have no competing interests.

## Authors' contributions

All authors of the paper have seen, reviewed and approved this manuscript. GEL and GAL were involved in all aspects of this paper. GEL and GAL shared in the conception and planning of the research project, conducted the statistical analyses and interpreted the findings. GEL wrote the method and results sections for this paper, RCG composed the introduction and reviewed draft versions of the manuscript. GEL and GAL collaborated on the discussion section of the paper. SH, AM, NC and RNW assisted with the conceptualization of the paper, coordination and data collection as well as the review of draft versions of the manuscripts.
